# Rate of USMLE Step 2 CK Scores Included on Orthopedic Surgery Applications and Associations With Step 1 Score

**DOI:** 10.7759/cureus.15008

**Published:** 2021-05-13

**Authors:** Stephen D Bigach, Daniel J Johnson, Joshua C Patt, Matthew D Beal

**Affiliations:** 1 Department of Orthopaedic Surgery, Northwestern University Feinberg School of Medicine, Chicago, USA; 2 Department of Orthopaedic Surgery, Carolinas Medical Center, Charlotte, USA

**Keywords:** medical education, usmle, step 2, orthopedic surgery, clinical knowledge scores

## Abstract

Background

At present, orthopedic surgery applicants do not universally include Step 2 Clinical Knowledge (Step 2 CK) scores on their applications and current inclusion rates are not yet reported. As Step 1 transitions to pass/fail scoring, we suspect more applicants will include Step 2 CK scores. We sought to identify what percentage of applications currently include Step 2 CK, if applicants who include Step 2 CK (Step 1+CK) score lower on Step 1 than those not including Step 2 CK (Step 1-CK), and what correlations exist between Step 1 and Step 2 CK scores among those who include the scores on their applications.

Methodology

Applicants to one residency program over two application cycles (2019-2020) were analyzed. The percentage including Step 1 and Step 2 scores was recorded. Step 1 scores were compared between Step 1+CK and Step 1-CK applicants. Differences between Step 2 CK and Step 1 scores were stratified by Step 1 score.

Results

A total of 1,688 applicants applied to our institution from 2019 to 2020. Of those reporting United States Medical Licensing Examination scores, 1,316/1,660 (79%) reported a Step 2 CK score. Step 1-CK applicants scored higher on Step 1 (250.7 ± 10.9) versus Step 1+CK applicants (244.3 ± 13.1) (p < 0.0001). More applicants who scored lower on Step 1 improved upon their percentile rank between Step 1 and Step 2 CK than those who scored higher on Step 1 (χ^2^_(8,1316)_ = 79.1, p < 0.0001).

Conclusions

From 2019 to 2020, 79% of applicants included Step 2 CK. Lower Step 1 scores were more likely to include Step 2 CK and improve upon their percentile score. It is unclear how Step 1 scoring change will affect current practice.

## Introduction

As a result of the Invitational Conference on United States Medical Licensing Examination (USMLE) Scoring (InCUS), the Federation of State Medical Boards and National Board of Medical Examiners announced that the USMLE Step 1 will be transitioning to pass/fail scoring no earlier than January 1, 2022 [1]. The impact of this decision on the resident selection process is at present unclear and may take years to fully appreciate. However, given that a significant number of Orthopedic Surgery Program Directors (PDs) have indicated that they currently use Step 1 as an application screening tool, ranging from 83% [2] to 94% [3], the impact of eliminating Step 1 scores will likely be profound. Cohn et al. [4] in their survey study of Orthopedic PDs noted that USMLE Step 2 Clinical Knowledge (Step 2 CK) is most likely to increase in importance with this change. Given this, we sought to evaluate the current practice of orthopedic applicants regarding inclusion of Step 2 CK on their applications. At present, the examination is not uniformly required, as Step 1 is, by most programs prior to application review, although some programs require passage prior to submission of final rank list.

The fourth year of medical school for orthopedic surgery applicants typically includes orthopedic surgery elective rotations at an applicant’s home program as well as away rotations at chosen programs of interest [5,6]. An increasing number of students have taken to performing a multitude of away rotations [5] to try and improve their rate of matching as residency PDs frequently rate the away rotation of high importance when formulating their final rank lists [4,7]. Sometime amid rotations, students are required to complete Step 2 of the licensure process including Step 2 CK and Step 2 Clinical Science (Step 2 CS). Those that serve as mentors to medical students interested in orthopedic surgery know that one of the most pressing questions from students is about timing of taking their Step 2 CK exam. Evidence suggests that taking the exam later in the year is associated with decreased scores [8] potentially related to the amount of time elapsed since completing core clerkships. However, some students choose to forgo taking Step 2 CK early enough to be on their application to focus on away rotations and allow their Step 1 score to stand alone. We hypothesized that students who do not submit their Step 2 CK score with their applications would have higher Step 1 scores, while those who included a Step 2 CK score would have lower Step 1 scores using Step 2 CK as a way to improve their application.

As a final component of this investigation, with the transition from Step 1 to pass/fail scoring, we evaluated if Step 1 scores were correlated with Step 2 CK scores and by how many points these scores typically differ. The exam scores are not a 1:1 conversion with a given percentile score represented by a higher three-digit Step 2 CK versus Step 1 score [9]. We suspected that our findings would be consistent with the literature showing Step 2 CK scores correlate significantly with Step 1 scores [10-12]. We were also interested in how scores varied grossly and by percentile rank from Step 1 to Step 2 CK.

## Materials and methods

After confirming exemption from our Institutional Review Board, we analyzed a de-identified convenience sample of USMLE scores of applicants to the Orthopaedic Surgery Residency Program at Northwestern University Feinberg School of Medicine. Data were made available from 2019 to 2020 application cycles for evaluation. Data from the 2019 cycle were compared against data from 2020 to identify any potential difference in applicant pools.

Step 1 scores from those applicants reporting Step 2 CK scores (Step 1+CK) and those not reporting Step 2 CK scores (Step 1-CK) were compared. Percentile and point differences between applicant Step 1 scores and Step 2 CK scores were calculated. As percentile scores were unavailable for direct comparison, we utilized mean and standard deviation (SD) data from Step 1 (2018) and Step 2 CK (2018-2019) from the USMLE score interpretation guidelines [9] to generate percentiles from a normal distribution. These applicants were then stratified by Step 1 scores and differences were analyzed.

Continuous variables are reported as mean ± SD. Categorical variables are reported as counts and percentages of whole. Continuous variables were tested for normality using Shapiro-Wilk test and compared with a Student’s t-test or Mann-Whitney U-test as appropriate. For comparison of multiple means, a post hoc Tukey test for multiple comparisons was performed. Categorical variables were compared with Pearson chi-square tests. Pearson coefficients were then used to evaluate correlation of Step 1 versus Step 2 CK scores as well as the difference between Step scores versus Step 1 score. Alpha level was set at 0.05. All data and statistical analyses were performed using JMP Pro (version 15.0, SAS, Cary, NC, USA).

## Results

Across the two years of study, 1,688 applications were received by the Northwestern University Orthopaedic Surgery Department for 12 total positions (six each year) at a 140:1 application-to-position ratio. This included 881 of the 1,192 (73.9%) total applicants reported by the National Residency Match Program (NRMP) in 2020 and 807/1,037 (77.8%) in 2019. Of the applications across both years, 1,660/1,688 (98.3%) reported a Step 1 score, 1,316/1,688 (78.0%) reported a Step 2 CK score, and 25/1,688 (1.5%) reported a Step 3 score. Percentages of applicants reporting Step scores by year are reported in Table 1. No significant differences were noted between the 2019 and 2020 applicant pools and thus statistics from the pooled data were used (Table 2). Of those applicants who included a Step 1 score, 79.3% (1,316/1,660) reported a Step 2 CK score. Overall, the average Step 1 score across all applicants was 245.7 ± 12.9. Step 1 scores for Step 1+CK applicants (244.3 ± 13.1) were significantly lower than Step 1-CK applicant scores (250.7 ± 10.9) (p < 0.0001).

**Table 1:** Percentages of step scores included by year. NRMP: National Residency Match Program; CK: Clinical Knowledge †Percentage of NRMP totals, *Percentages of step scores taken from Northwestern University application totals

	Applications (NRMP totals)	Applications^†^ (Northwestern)	Step 1^*^	Step 2 CK^*^	Step 3^*^
Combined	2,229	1,688 (75.7%)	1,660 (98.3%)	1,316 (78.0%)	25 (1.5%)
2020	1,192	881 (73.9%)	872 (99.0%)	700 (79.5%)	11 (1.3%)
2019	1,037	807 (77.8%)	788 (97.6%)	616 (76.3%)	14 (1.7%)

**Table 2:** Applicant scores by year. CK: Clinical Knowledge

	Combined	2020	2019	2019 versus 2020
Step 1	245.7 ± 12.9 (N = 1,660)	245.4 ± 12.8 (N = 872)	245.9 ± 13.1 (N = 788)	p = 0.398
Step 1+CK	244.3 ± 13.1 (N = 1,316)	244.3 ± 12.8 (N = 700)	244.5 ± 13.4 (N = 616)	p = 0.713
Step 1-CK	250.7 ± 10.9 (N = 344)	250.2 ± 11.5 (N = 172)	251.2 ± 10.2 (N = 172)	p = 0.583
Step 2	252.8 ± 12.8 (N = 1,316)	252.5 ± 12.5 (N = 700)	253.3 ± 13.2 (N = 616)	p = 0.160

The average Step 2 CK score across 2019-2020 was 252.8 ± 12.8. On average, Step 1+CK applicants scored 8.5 ± 10.5 points higher on Step 2 CK but decreased by 4.6 ± 18.4 percentage points; however, this varied significantly based on Step 1 score, as shown in Table 3 (χ^2^_(8,1316)_ = 79.1, p < 0.0001). Post hoc testing results shown in Table 4 demonstrate that applicants scoring <215 on Step 1 demonstrated greater percentile increase on Step 2 CK than all applicants scoring more than 225 (p < 0.0001-0.0029), and likewise applicants scoring between 215 and 224 on Step 1 had significant increases versus those scoring between 235 and 264 (p < 0.0001-0.0021). In total, when compared with their Step 1 scores, 271/1,316 (20.6%) of applicant’s raw three-digit Step 2 CK scores did not improve or worsen while an additional 494/1,316 (37.5%) applicants improved upon their three-digit score from Step 1 but their percentile decreased between the exams. Thus, in total, only 551/1,316 (41.9%) of applicants achieved a percentile on Step 2 CK equal to or better than their percentile achieved on Step 1.

There was a strong correlation between Step 1 and Step 2 CK scores (r = 0.675, p < 0.0001) in this study with 45.6% of Step 2 CK score variance explained by an applicant’s Step 1 score. Linear regression modeling generated the following model:

$Step 2 CK = 0.661 &times; Step 1 Score + 91.29$

**Figure 1:**
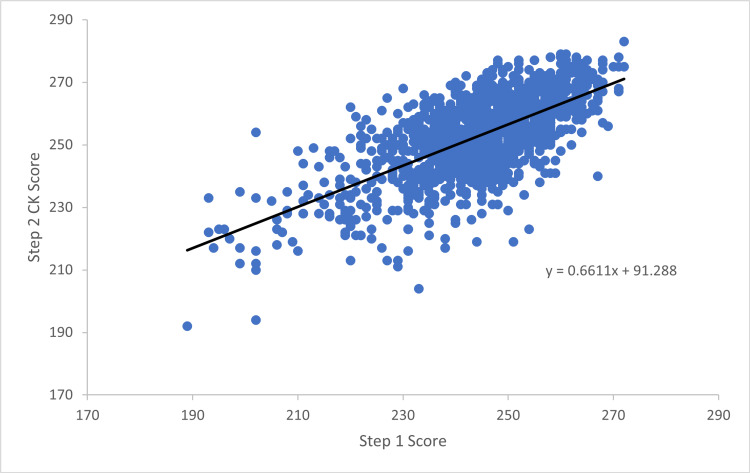
Step 2 CK score versus Step 1 score. Step 2 CK scores graphed as a function of Step 1 score with a line of best fit. There was a strong correlation between Step 1 and Step 2 CK in this study (r = 0.675, p < 0.001) CK: Clinical Knowledge

**Figure 2:**
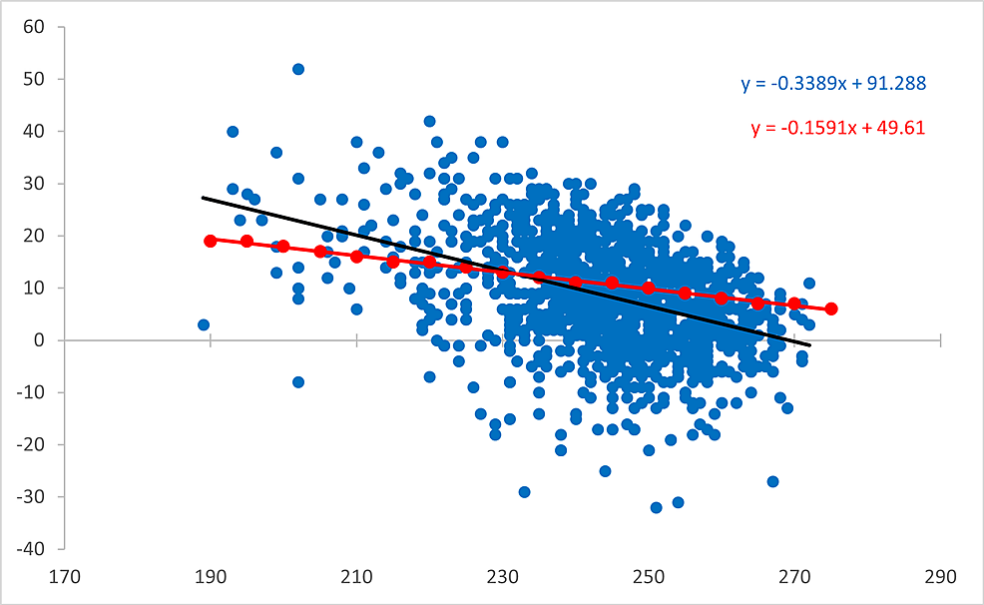
Difference between Step 2 CK and Step 1 scores versus Step 1 score. Differences from Step 2 CK score to Step 1 score graphed as a function of Step 1 scores. There was a moderate correlation between score difference and Step 1 score (r = 0.425, p < 0.001). Graphed in red are the expected score differences calculated by percentile achieved on Step 1 using the mean, standard deviation data from 2018 (Step 1) and 2018-2019 (Step 2 CK) score interpretation guidelines [9]. The lines of best fit for observed (black) and expected (red) values intersect at a Step 1 score of 231.8 indicating the score where applicants begin scoring below their percentile achieved on Step 1 CK: Clinical Knowledge

The correlation is demonstrated in Figure 1. Differences between Step scores had a moderate correlation with Step 1 score (r = 0.425, p < 0.0001) with a trend toward higher performers on Step 1 scoring lower than their previously achieved percentile and vice versa (Figure 2, Table 3). The intersection point between lines of best fit for expected difference based on percentile scores and actual difference appreciated occurred at a Step 1 score of 231.8.

**Table 3:** Step 2 score stratified by Step 1 score. *Percentiles calculated using means and standard deviations from 2018 for Step 1 (230 ± 19) and 2018-2019 for Step 2 CK (243 ± 16) CK: Clinical Knowledge

Step 1 Score	N	Mean point difference	Mean % difference^*^	Step 2 CK % ≥ Step 1 %^*^
265+	39	1.1 ± 7.3	-5.0% ± 9.3%	12 (30.8%)
255-264	254	3.9 ± 7.5	-6.7% ± 10.8%	74 (29.1%)
245-254	410	5.9 ± 9.4	-9.9% ± 17.1%	138 (33.7%)
235-244	370	10.7 ± 9.5	-3.3% ± 19.3%	184 (49.7%)
225-234	142	13.6 ± 11.7	2.2% ± 23.2%	82 (57.7%)
215-224	62	16.2 ± 11.6	6.1% ± 23.7%	33 (53.2%)
<215	39	21.5 ± 11.2	10.2% ± 16.8%	28 (71.8%)

**Table 4:** Significance values for percentile increases from Step 1 to Step 2 CK as stratified by Step 1 score. p-values from post hoc Tukey analysis with p < 0.05 considered significant CK: Clinical Knowledge

	<215	215-224	225-234	235-244	245-254	255-264	265+
<215	-	0.915	0.157	0.0001	<0.0001	<0.0001	0.003
215-224	-	-	0.777	0.002	<0.0001	<0.0001	0.036
225-234	-	-	-	0.026	<0.0001	<0.0001	0.271
235-244	-	-	-	-	<0.0001	0.227	0.998
245-254	-	-	-	-	-	0.278	0.654
255-264	-	-	-	-	-	-	0.998
265+	-	-	-	-	-	-	-

## Discussion

The main findings of this study are (1) 79.3% of orthopedic surgery residency applications in 2019 and 2020 included both Step 1 and Step 2 CK scores; (2) those who do not report their Step 2 CK scores on average have higher Step 1 scores; (3) percentile differences between Step 1 and Step 2 CK scores vary significantly by Step 1 score; and (4) a strong correlation exists between Step 1 and Step 2 CK scores with 45.6% of Step 2 CK score variance explained by Step 1.

As the landscape of resident selection changes in response to Step 1 scoring transitioning to pass/fail, it will be vitally important to understand the ramifications of these changes on orthopedic surgery applicants and programs alike. The purpose of this study was to establish a baseline understanding of applicant behavior in the fourth year as it pertains to standardized testing, which included measuring the current percentages of students who include the USMLE Step 2 CK on their applications and how they perform on Step 2 CK relative to their Step 1 performance. Li et al. [3] reported that the percentage of programs reporting a target Step 2 CK score to the NRMP increased from 41.8% in 2008 to 50% in 2016 while Step 1 simultaneously increased from 79.5% to 94%. The rise in these numbers, which the authors concluded was attributable to the rise in applications, suggests an increasing importance and utilization of USMLE scores. In fact, changes such as these and arguments that standardized testing does not accurately represent what programs should be looking for in applicants are part of the call for reform that lead to the InCUS decision [1,13,14]. Nonetheless, as Step 1 transitions to pass/fail and Step 2 CK remains numerically scored, Step 2 CK seems poised to rise in importance, at least according to early survey reports from Orthopedic PDs [4]. Because of this, we suspect the inclusion of Step 2 CK on applications will increase from 79.3% found in the present study. For this to occur, more students would need to take the examination earlier in fourth year. As the importance of the test score increases, this may have a trickle-down effect on the scheduling of away rotations for all students, but perhaps more so for weaker applicants who have been reported to attend more away rotations in hopes of improving their chance to match [5]. Further research will be needed to delineate this after the score reform.

Our hypothesis that Step 1-CK applicants score higher on Step 1 is supported by the present study with these applicants outscoring Step 1+CK applicants by approximately 6.5 points. This suggests that Step 1-CK applicants are satisfied with their Step 1 scores and feel confident in their applications. This may indicate that they prioritize other aspects of their fourth-year education including electives and away rotations. More research would be needed to identify if this is in fact the case. For example, evaluating the number of programs Step 1-CK applicants apply to or the number of rotations they perform may be an indicator as smaller numbers for both have been linked to perceived application strength [15]. Secondarily, we were interested to see if Step 1+CK applicants performed better than expected compared to their Step 1 examinations, perceiving the second score as an opportunity to improve upon a disappointing effort on Step 1. Although this did not appear to be the case overall, with an average percentile change of -4.6, when differences were stratified by Step 1 score, applicants who scored lower on Step 1 were more likely to improve upon percentile achieved than those who scored higher on Step 1. Multiple possible explanations exist for this phenomenon including a ceiling effect for those test takers who scored sufficiently high on Step 1, the statistical variability associated with the USMLE examinations [9], or students with lower Step 1 scores were more motivated than those with higher scores to study for Step 2 CK, ultimately improving their performance compared to Step 1. As multiple studies have shown that scoring well on standardized tests predicts future standardized test success [10,12,16], the discrepancy between scoring differences reported in the present study suggests an element of effect modification or confounding or both. Importantly, the scoring change of Step 1 may affect both factors. If the discrepancy can be explained by statistical variability of scoring, or confounding, the Step 1 scoring change will eliminate a second data point that at present allows for a clearer and earlier picture of applicant proficiency. Perhaps equally as important, if the discrepancy is related to applicant motivation, or effect modification, with increased focus on Step 2 CK we suspect this would be eliminated. In other words, with a more “high stakes” Step 2 CK exam, we suspect those high scoring Step 1 applicants will perform better on Step 2 CK. Medical students have proven that they will spare no expense or resource to impress residency PDs and secure a position in the match [17-19], with Step 2 CK remaining scored, we can expect medical student focus to shift toward this exam.

One limitation of this study is the use of a convenience sample from one residency program’s records as opposed to the entire NRMP sample. However, given that this sample contains 1,688/2,229 (75.7%) of all NRMP applications over this period, it is unlikely that results would change significantly if evaluating the entire sample. Although it is certainly possible that applicants applying to Northwestern University included Step 2 CK at significantly different rates than applicants not applying to Northwestern University. Our program’s website at the time of study did not specifically comment on inclusion of Step 2 CK scores but noted requirement of “USMLE Scores” [20]. Another limitation is the comparison of percentile scoring between Step 1 and Step 2 CK. Percentile scores were calculated using the most recently reported means from the USMLE scoring guidelines [9]. Because we did not have the year that each applicant took their Step 1 or Step 2 CK examinations, we could not utilize the specific yearly population mean and standard deviations reported by the USMLE. Thus, changes in mean and SD across examination years could influence our calculations but changes are relatively minimal [9]. And finally, this study is only able to report current practice of orthopedic surgery residency applicants, and suggest theoretical changes to medical student practices, but future research will be needed to assess if indeed more orthopedic surgery applicants include Step 2 CK scores on their transcripts moving forward.

## Conclusions

This study indicates that at the present time, 79% of orthopedic surgery applicants are including Step 2 CK scores on their applications. These applicants score lower on Step 1 than those who do not include Step 2 CK. While only 42% of applicants maintained or improved upon their percentile score from Step 1 to Step 2 CK, when stratified by Step 1 scores, a much higher percentage of applicants with lower Step 1 scores showed improvement. It remains to be seen if and how the transition to pass/fail Step 1 scoring will change current practices.
